# Genetic Evidence for the Role of a Rice Vacuolar Invertase as a Molecular Sink Strength Determinant

**DOI:** 10.1186/s12284-018-0201-x

**Published:** 2018-01-17

**Authors:** Shamitha Rao Morey, Tatsuro Hirose, Yoichi Hashida, Akio Miyao, Hirohiko Hirochika, Ryu Ohsugi, Junko Yamagishi, Naohiro Aoki

**Affiliations:** 1 Graduate School of Agricultural and Life Sciences, The University of Tokyo, 1-1-1 Yayoi, Bunkyo-ku, Tokyo, 113-8657 Japan; 2 Central Region Agricultural Research Center, NARO, 1-2-1 Inada, Joetsu, Niigata 943-0193 Japan; 3 Advanced Genomics Breeding Section, Institute of Crop Science, NARO, 2-1-2, Kannondai, Tsukuba, Ibaraki 305-8518 Japan; 4 Present addresses: Research Institute for Food and Agriculture, Ryukoku University, 1-5 Yokotani, Seta Oe-cho, Otsu, Shiga 520-2194 Japan

**Keywords:** Vacuolar invertase, Grain size, Cell expansion, Hexose-to-sucrose ratio, Grain weight, Sink strength, Assimilate partitioning

## Abstract

**Background:**

Rice is a major crop feeding the majority of the global population, and increasing its sink strength is one of the modes to alleviate the declining availability of food for the rapidly growing world population. We demonstrate a role for an important rice vacuolar invertase isoform, OsINV3, in sink strength determination.

**Results:**

*OsINV3* mutants showed shorter panicles with lighter and smaller grains, owing to a smaller cell size on the outer and inner surfaces of the palea and lemma as observed by scanning electron microscopy. Further, strong *promoter::GUS* expression was observed in the palea, lemma and the rachis branches in the young elongating panicles, which supported the role of *OsINV3* in cell expansion and thus, in spikelet size and panicle length determination. Size of the spikelet was found to directly influence the grain weight, which was confirmed by the lack of differences in weights of hulled grain for differently segregated alleles in the heterozygous lines. Assessment of field grown mutants not only revealed a drastic reduction in the percentage of ripened grain, 1000-grain weight and final yield, but also significantly reduced partitioning of assimilates to the panicles, whereby the total dry weight remained unaffected. Determination of the non-structural carbohydrate contents revealed a lower hexose-to-sucrose ratio in the panicles of the mutants from panicle initiation to 10 days after heading, a stage that identifies as the critical pre-storage phase of grain filling, whereas the starch contents were not affected. In addition, strong *promoter::GUS* expression was observed in the dorsal end of ovary during the pre-storage phase until 6 days after flowering, highlighting a function for OsINV3 in monitoring the initial grain filling stage.

**Conclusions:**

OsINV3 was found to regulate spikelet size by playing a key role in cell expansion, driving the movement of assimilates for grain filling by modulating the hexose-to-sucrose ratio, contributing in grain weight determination and thus, the grain yield.

**Electronic supplementary material:**

The online version of this article (10.1186/s12284-018-0201-x) contains supplementary material, which is available to authorized users.

## Background

Carbon that is assimilated by plants through photosynthesis is partitioned among various tissues based on their competitive ability to import assimilates. This ability is commonly termed as sink strength and has been previously described to be dependent on physical as well as physiological capacity of the sinks (Ho 1988; Marcelis 1996; Herbers and Sonnewald 1998; Bihmidine et al. 2013). While sink size determines the physical capacity, sink activity involving assimilate transport, utilization and storage by the sink cells, determines the physiological capacity (Ho 1988; Herbers and Sonnewald 1998). An increase in sink strength directly translates to an increase in yield, mainly due to higher assimilate partitioning into the sinks co-ordinated by an increased sink size. With respect to rice (*Oryza sativa* L.), an important cereal crop that feeds a majority of the global population, there is a pressing need to increase the sink strength in order to achieve global food security and enable sustenance by ensuring food for the rapidly growing population.

Cleavage of sucrose, a major photoassimilate in plants, plays an important role in carbon allocation, not only by osmotically controlling the flux, but also by generating hexose-based signals to regulate genes involved in carbon metabolism in the sink organs (Herbers and Sonnewald 1998; Smeekens 2000; Koch 2004). The enzymes that thus, catalyze sucrose cleavage are considered to be potential molecular contributors to sink strength (Herbers and Sonnewald 1998). One such class of enzymes are the invertases (EC 3.2.1.26) that catalyze the irreversible cleavage of sucrose into glucose and fructose. Based on their sub-cellular localization, solubility and optimum pH of function, they are classified into insoluble acid (cell wall), soluble acid (vacuolar) and soluble neutral (cytosolic) invertases. The entry of sucrose into different utilization pathways is regulated by various isoforms of invertases (Sturm 1999).

In rice, eight neutral invertases (NINs), nine cell wall invertases (CWINs) and two vacuolar invertases (VINs) have been identified (Ji et al. 2005). Till date, CWINs have been found to play a primary role in assimilate partitioning, thus regulating the grain weight in crops including rice (Cheng et al. 1996; Hirose et al. 2002; Wang et al. 2008; Tang et al. 2017; Li et al. 2013). For example, two rice CWIN isogenes, *OsCIN1* and *OsCIN2*, play a role in determination of sink strength by regulation of assimilate partitioning, where *OsCIN1* was found to play a role during the early grain filling stages, and *OsCIN2* in the regulation of grain size and weights (Hirose et al. 2002; Wang et al. 2008).

In the past, vacuolar invertases (VINs) have been attributed to roles in cell elongation of seedling hypocotyls in Arabidopsis (Sergeeva et al. 2006), fiber cell elongation in cotton (Wang et al. 2010), and rapidly expanding tissues in carrot taproot (Tang et al. 1999) and sugar beet petioles (González et al. 2005). It has been suggested that VIN regulates the sink size by driving cell expansion, owing to a turgor generated by influx of osmotic solutes in response to an increase in hexoses (Koch 2004; Sergeeva et al. 2006). It has also been considered that VINs drive cell expansion based on availability of carbohydrates (Koch 2004). Further, role of VINs in determination of hexose-to-sucrose ratio in potato (Zrenner et al. 1996) and tomato (Klann et al. 1996) suggests the importance of sink sugar composition in regulation of the sink size (Klann et al. 1996).

Roles of CWINs as contributors to sink strength, exhibiting a role in phloem unloading, have been extensively studied (Miller and Chourey 1992; Weber et al. 1995), and a CWIN *OsCIN2 (GIF1)* has been previously reported to play a key role in grain filling by affecting sucrose import by sinks (Wang et al. 2008). However, roles of vacuolar invertases in terms of sucrose import by sinks has not been a research focus in recent times. The present study examines the physiological role of a VIN isogene, *OsINV3,* in rice, in terms of sink size and assimilate partitioning, in concert with analysis of cell size and sink sugar composition, thus, establishing evidence for its role as a molecular sink strength determinant.

## Results

### Isolation of the Gene-Disruption Mutant of *OsINV3* and Phenotypic Analysis with Respect to Panicle Height, Grain Size and Grain Weight

**Fig. 1:**
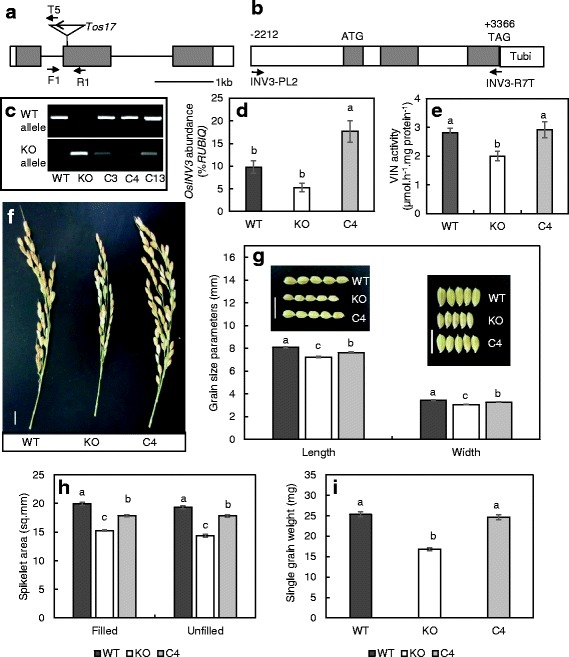
Characterization and phenotypic analysis of the mutants (KO). **a** *OsINV3* gene in 5′-3' direction with the *Tos17* insertion in the second exon in reverse orientation. Grey boxes denote exons separated by lines that denote introns, white boxes indicate 5′(left) and 3′(right) UTR, and arrows indicate primers F1, R1 and T5, **b** The construct used for complementation test containing the ORF of *OsINV3* along with 2212 bp upstream of the transcription start site, grey boxes indicating the exons, and arrows indicate primers INV3-PL2 and INV3-R7T, **c** Gel data showing the presence of WT and KO alleles in WT, KO and complement lines, **d** mRNA transcript level of *OsINV3* relative to *RUBIQ* (*n* = 3) and **e** Vacuolar invertase activity expressed as Vmax (*n* = 5), in mature leaf of WT, KO and C4, **f** Panicles from WT, KO and C4 lines, **g** Grain length and width parameters for WT, KO and C4 (*n* = 12), **h** Spikelet area for filled and unfilled spikelets from WT, KO and C4 lines (*n* = 12), **i** average weight per grain, for WT, KO and C4 lines (*n *= whole grain set from 3 plants). Data represent the mean ± SE. Same letters indicate insignificant differences between the lines tested using ANOVA (Tukey’s test) with significance level of *p* < 0.05. Vertical white bars indicate 1 cm

Following screening of approximately 40,000 lines with *Tos17* retro-transposon insertions, a mutant line NG6441 bearing a *Tos17* insertion within the *OsINV3* gene was isolated. Sequence analysis revealed that the *Tos17* retrotransposon in NG6411 was introduced in the second exon (Fig. 1a). The gene-disruption mutants segregated from NG6441 and the WT were grown under controlled conditions to assess major phenotypic differences. Differences in the observed traits were reconfirmed through complementation. The size of the amplicon indicating the allele carrying WT gene was 350 bp, and for that of mutant was 315 bp. Genotyping of the WT, mutant and the complementation lines C3, C4 and C13 confirmed a homozygous insertion of the *Tos17* retrotransposon in the second exon of the *OsINV3* gene in the mutants, and subsequent successful incorporation of *OsINV3* gene in the mutant lines, enabling an effective complementation (Fig. 1c). The mRNA transcript levels revealed through real-time PCR showed an overexpression in the C4 line (Fig. 1d). The presence of aberrant transcript in the mutants was reconfirmed by semi-quantitative PCR using primers F5 and R5 spanning the *Tos17* insertion, that showed an absence of amplicon in the mutants (Additional file 1: Figure S1A), while primers F6 and R6 targeting the region downstream of the *Tos17* insertion showed a presence of amplicon, however, lighter in intensity (Additional file 1: Figure S1B), coinciding with the real-time data (Fig. 1d). The enzyme activity in the mature leaves of the mutant lines was lower by 28.8% relative to the WT, suggesting a reduction in soluble acid invertase activity in the mutants, with observed recovery in the C4 line (Fig. 1e). It was observed that the panicles of the mutants were shorter than that of the WT, with displayed recovery of the trait upon complementation (Fig. 1f). Grain length and width of unhulled grain was also smaller in the mutant when compared with the WT, with significant recovery observed for both the traits in C4 (Fig. 1g). Similar results were obtained for the area of both filled and unfilled spikelets, where smaller area of the mutants was significantly recovered in the C4 lines (Fig. 1h). In addition to smaller grain size and panicle height, average single grain weight was also lower in the mutant lines, displaying an absolute recovery for the trait in C4 (Fig. 1i). These results indicate a significant role for *OsINV3* in the regulation of panicle height, grain size and grain weight.

**Fig. 2:**
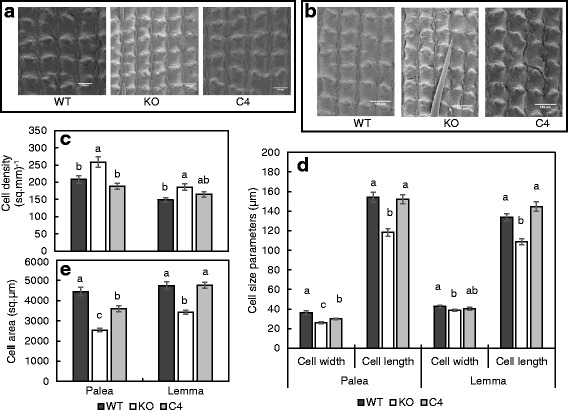
Spikelet cell number and size analysis using SEM. Cell density for a fixed area in the outer surface of **a** palea and **b** lemma for WT, mutant (KO) and C4. **c** Cell density expressed as number of cells/sq.mm of the outer surface of palea and lemma for WT, KO and C4. **d** Cell size parameters, depicting cell width and cell length and **e** Cell area in sq.μm, for the inner surface of palea and lemma. Data represent the mean ± SE (*n* = 9). Same letters indicate insignificant differences between the lines tested using ANOVA (Tukey’s test) with significance level of *p* < 0.05. Horizontal bars represent 100 μm

In order to isolate the role of *OsINV3* in spikelet cell expansion, spikelets at 2 days before heading (DBH) from WT, mutant and the C4 lines were analyzed using scanning electron microscopy (SEM). The cell density in mutant on the outer surface of both palea and lemma were significantly higher than the WT, with observed recovery in the C4 line (Fig. 2a–c). The cells were stacked closer in the mutants when compared to the WT and the C4 lines. For an observed 23.5% decrease in spikelet area (Fig. 1h), a 24.3% increase in the cell density (Fig. 2c) was established suggesting a possible lack of difference in cell number. However, cell size on the outer surface of the spikelets of mutant was considerably smaller (Fig. 2a and b). The epidermal cells on the inner surface of palea and lemma were analyzed, with decreased cell area, cell width and cell length in the mutant, and recovery for these traits in the C4 lines (Fig. 2d and e). Although absolute recovery in the cell length, and cell area in the lemma of C4 was observed; cell width and cell area in the palea, although significant, were only partially recovered. Thus, the role of *OsINV3* in cell expansion in regulation of spikelet size was established.

### Grain Weight Distribution for Segregated Alleles in the Heterozygous Lines

To explore whether the small grain phenotype is caused by disruption of *OsINV3* in the maternal or the filial tissues, seeds borne on the heterozygous (+/−) plants were analyzed, and corresponding weights of hulled grain were determined for the segregated alleles, namely INV3 +/−, INV3 +/+ and INV3 −/−. A lower grain weight as observed in the mutant (−/−) plant was not the case in INV3 −/− grain borne on the heterozygous lines (Table 1). Lack of differences in the grain weight of seeds on INV3 +/− lines irrespective of the genotype indicates that the small grain phenotype of the mutant (−/−) plant is not solely caused by the filial factor (highlighting a maternal influence on grain weight).

**Table 1:** Distribution of grain weights for the WT and mutant (KO) alleles segregated in the heterozygous lines

Genotype of plant	Plant Number	Genotype of seeds	N	Single grain weight (mg)
Heterozygous (+/−)	1	INV3 +/+	11	22.0 ± 2.3
		INV3 +/−	15	22.9 ± 2.7
		INV3 −/−	6	21.6 ± 1.3
	2	INV3 +/+	9	22.7 ± 2.0
		INV3 +/−	16	22.4 ± 1.5
		INV3 −/−	7	22.0 ± 2.0
	3	INV3 +/+	8	21.7 ± 2.0
		INV3 +/−	17	21.4 ± 1.7
		INV3 −/−	7	21.3 ± 2.0
WT (+/+)		INV3 +/+	32	22.1 ± 1.6
KO (−/−)		INV3 −/−	32	14.0 ± 1.5

Seeds borne on heterozygous (+/−) plants were individually weighed and genotyped to determine the filial regulation of grain weights. Note that grain weight differences were absent between the segregated alleles on +/− plants, while were found to be significant between +/+ and −/−. Data represent the mean ± standard deviation

### Expression of *OsINV3* During Various Stages of Panicle Development

**Fig. 3:**
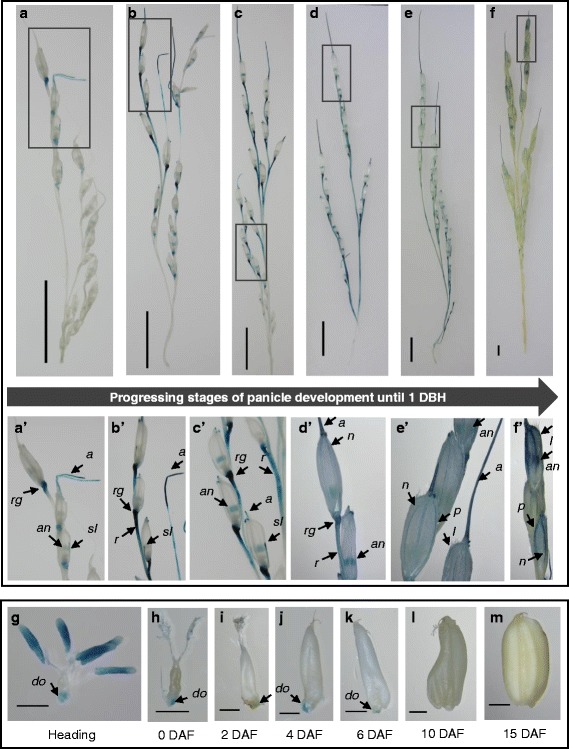
Spatio-temporal expression of *OsINV3* in the panicle using *promoter::GUS* lines. **a**-**f** GUS stained panicles at different progressing stages of panicle development until 1 DBH; **a'**-**f'** magnified images of the area marked in **a**-**f**. **g**-**m** *Promoter::GUS* expression in the developing endosperm at heading, 0 DAF, 2 DAF, 4 DAF, 6 DAF, 10 DAF and 15 DAF respectively. Vertical bars indicate 1 cm and horizontal bars indicate 1 mm. Arrows show the areas stained, isolating a vascular trace of *OsINV3* expression in rachis branches (*r*)*,* palea (*p*), lemma (*l*), nerves (*n*), anther (*an*), sterile lemmas (*sl*), rudimentary glume (*rg*), awn (*a*) and the dorsal end of the ovary (*do*)

*OsINV3 promoter:GUS* assay for panicles at progressing stages of panicle development were studied to isolate the temporal and spatial expression pattern in the panicles. Early panicle development stages of young panicles, 0.5 cm – 1 cm in length, showed no GUS activity except for in the bract hair (Additional file 1: Figure S2A). Expression of *OsINV3* was first observed in panicles ~ 3 cm in length, in the rudimentary glume, pedicel and the empty glumes in the spikelets in upper position (Fig. 3a and a'), following a similar expression pattern in the spikelets in lower position along the progressing stages of development. The expression in rachilla and spikelet was observed basipetally along the progressing stages of panicle development (Fig. 3b-f, 3b'-f'). A strong staining in the anthers was also observed in all stages (Fig. 3b'-f'), showing expression in the epidermal cells as well as the pollen (Additional file 1: Figure S2D). Strong expression was observed in the dorsal end of the ovary following fertilization until 6 days after flowering (DAF) (Fig. 3g-k). However, no expression in the developing grain was observed following this stage (Fig. 3l and m).

### Assessment of Agronomic Traits of the Field Grown Mutants

**Fig. 4:**
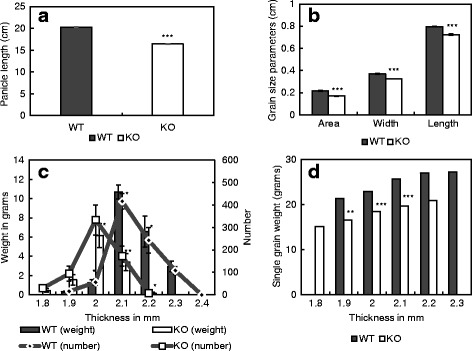
Panicle and grain size analysis for field grown mutants (KO). **a** Panicle length (*n* = 29), **b** grain size parameters of unhulled grain (*n* = 30), **c** grain number and weight for corresponding grain thickness, **d** single grain weight for corresponding grain thickness are shown. Data represent the mean ± SE (*n* = 3 plants). Asterisks indicate statistical significance of difference using Student’s T-statistic with *-*p* < 0.05, **-*p* < 0.01 and ***-*p* < 0.001

Field trials for the *OsINV3* WT and the mutant were performed to test their agronomic and growth characteristics. Not only were the grains of the mutant smaller (Fig. 4b), the yield, grain weight and the percentage of ripened grain were also significantly lower in the mutant when compared to the WT, with 17.8%, 30.3% and 47.9% reduction in the percentage of ripened grain, 1000-grain weight and yield respectively (Table 2).

**Table 2:** Yield and its components for WT and mutant (KO)

	Number of productive panicles	Number of spikelet per panicle	Percentage of ripened grain	Grain weight (g 1000 grains^− 1^)	Yield (g m^− 2^)
WT	9.3 ± 0.4	112.4 ± 2.1	76.6 ± 2.8	27.7 ± 0.1	491.8 ± 25.2
KO	9.2 ± 0.4	107.8 ± 2.8	63 ± 2.2***	19.3 ± 0.1***	256.2 ± 18.7***
% reduction	n.s.	n.s.	17.75	30.32	47.91

Data represent the mean ± SE (*n* = 12 plants). Asterisks indicate statistical significance of difference using Student’s T-statistic with ***-*p* < 0.001. Results of the 2016 field experiment indicated, with similar results obtained in 2015

The field grown WT and the mutants for *OsINV3* reconfirmed the phenotype of shorter panicles and smaller grains as observed in controlled conditions. The mutant panicles were significantly shorter (Fig. 4a), however, with no differences in the number of primary or secondary rachis branches when compared to the WT (Additional file 1: Figure S3A). The mutant grains, both unhulled and hulled were smaller in size than the WT (Additional file 1: Figure S3B). The length, width and area of filled grains were all significantly smaller for the mutant when compared to the WT (Fig. 4b). Further, the distribution of grain number (Fig. 4c) and grain weight for a corresponding grain thickness revealed a significantly shorter peak and a smaller area under the distribution curve for the mutant, with the single grain weight for a corresponding grain thickness significantly smaller in the mutants (Fig. 4d).

**Fig. 5:**
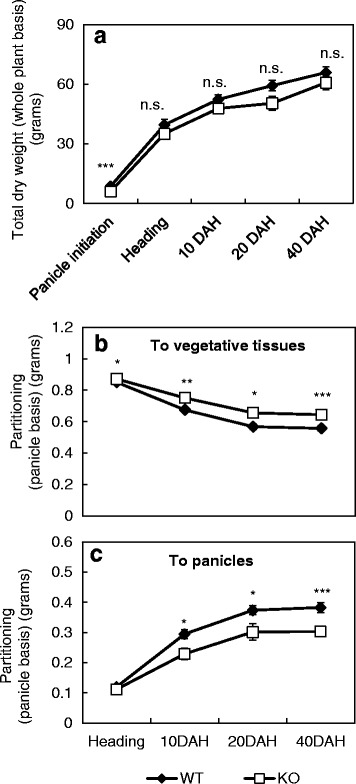
Dry matter weight differences between the WT and the mutants (KO). **a** Total dry weights measured on whole plant basis, **b** Dry matter partitioning to the vegetative tissues and, **c** panicles, on per panicle basis for *OsINV3* WT and KO. Data represent the mean ± SE (*n* = 9). Asterisks indicate statistical significance of difference using Student’s T-statistic with *- *p* < 0.05, **-*p* < 0.01 and ***-*p* < 0.001; and n.s. – not significant. X-axis – representative of stage, and not scaled to number of days

Dry weight partitioning is considered to be the end result of assimilate partitioning in plants (Marcelis 1996). Hence, total dry weight, per plant was determined for the WT and mutant at different stages from panicle initiation to harvest (Fig. 5a) in order to estimate possible differences in assimilate partitioning. The differences that existed at panicle initiation (with a lower plant dry weight for the mutant), ceased to exist at all other stages. Given, the lower grain weight of the mutant (Table 1) at harvest, this lack of total dry weight difference between the WT and the mutant at the grain filling stages indicates differences in assimilate partitioning between panicles and other tissues. As conceived, the dry matter partitioning to panicles was significantly lower in the mutant at all the grain filling stages (Fig. 5c), while the dry matter partitioning to vegetative tissues was significantly higher in the mutant when compared to the WT (Fig. 5b).

Further, the photosynthetic capacity of the WT and mutant at panicle initiation, heading and late ripening were not different (Additional file 1: Figure S4), displaying a maintained source ability. Thus, it could be conceptualized that for a given amount of carbon fixed by the WT and mutant plants, the percentage of assimilate partitioned into sinks (panicles) was indeed lower in the mutants, suggestive of a lower sink ability to import photoassimilates in the vacuolar invertase mutants.

### Analyses of Non-Structural Carbohydrate (NSC) Contents During the Period of Panicle Development and Grain Filling

**Fig. 6:**
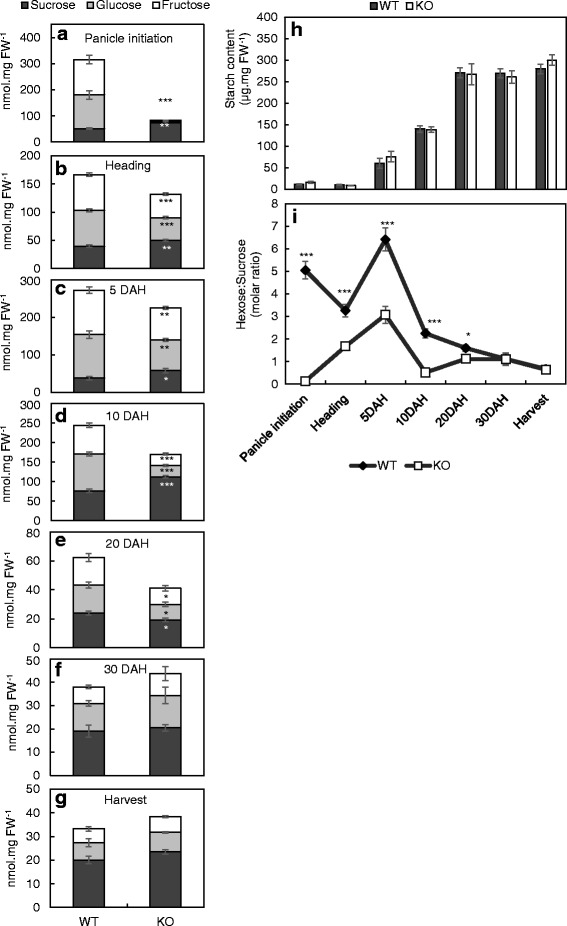
**a**-**i**: NSC profile of panicles of the WT and the mutant (KO) during growth stages from panicle initiation to harvest. **a-g**: Concentration of sugars in the panicle at panicle initiation, 5 DAH, 10 DAH, 20 DAH, 30 DAH and harvest; **h**: Starch content in the panicles; **i**: Hexose-to-sucrose ratio in the panicles of WT and the mutant at similar growth stages as mentioned above. Data represent the mean ± SE (*n* = 6). Asterisks indicate statistical significance of difference using Student’s T-statistic with *-*p* < 0.05, **-*p* < 0.01 and ***-*p* < 0.001. X-axis – representative of stage, and not scaled to number of days

The amounts of NSCs in the whole panicles of field grown plants were estimated at stages from panicle initiation to maturity (Fig. 6). While levels of starch in both the WT and the mutant were not different (Fig. 6h), significant differences in the sugar levels were determined till 20 days after heading (DAH) (Fig. 6a-e), where the mutant showed higher sucrose and lower hexose levels until 10 DAH (Fig. 6a-d). At 20 DAH, sucrose and hexoses were found to be lower in the mutant than in the WT (Fig. 6e). After this stage, no differences between the WT and the mutant were observed until maturity (Fig. 6f and g). The differences in the sugar composition were reflected in the hexose-to-sucrose ratios, where, although the trend was maintained from heading to harvest for both WT and mutants, the ratio was significantly lower for the mutants between heading to 10 DAH, which signify the pre-storage phase of grain filling (Fig. 6i). However, a stark difference in hexose-to-sucrose ratio between the WT and the mutant was observed at panicle initiation stage (Fig. 6i) usually characterized by high hexose levels, owing to a higher sucrose and negligible hexose levels in the mutants (Fig. 6a). The vacuolar invertase activity levels were however, not different between the WT and mutant for young panicles at this stage (Additional file 1: Figure S5A), nor were the neutral (Additional file 1: Figure S5B) and cell wall invertase activities (Additional file 1: Figure S5C). Absence of differences in vacuolar invertase activity between the WT and mutant could be attributed to functional redundancy exhibited by *OsINV2*, the transcript levels of which were significantly higher in the mutants when compared to the WT (Additional file 1: Figure S5D).

## Discussion

The present study characterizes one of the isoforms of vacuolar invertase genes, *OsINV3* in rice and determines its physiological roles in terms of sink expansion and partitioning into the sinks.

### Key Role of OsINV3 in Determination of Spikelet Size and Its Influence on Grain Weight

The *OsINV3* mutants demonstrated smaller sinks by displaying shorter panicle and smaller grain phenotypes, owing to smaller cell size in both the inner and outer surfaces of palea and lemma of the mutant spikelets as demonstrated by the SEM analysis (Fig. 2). Role of vacuolar invertases in cell expansion by generation of a turgor in the cell has previously been suggested in Arabidopsis roots (Sergeeva et al. 2006), developing petioles and primary roots of carrot (Sturm et al. 1995) and has been widely accepted (Tang et al. 1999; Roitsch and González 2004; González et al. 2005; Ruan et al. 2010). The expression of *OsINV3* in the spikelet as observed in the *promoter::GUS* lines in the palea, lemma and the nerves during the panicle development stages (Fig. 3d-f, 3d'-f') and the early grain filling stages (Additional file 1: Figure S2B), is consistent with the role of *OsINV3* in cell expansion in the spikelet. Size of the spikelet plays a crucial role in determination of final grain weight (Wang et al. 2008; Song et al. 2015), mainly by modifying the grain filling rate, eventually affecting the grain weight (Wang et al. 2008). Absence in individual grain weight differences for grains borne on the heterozygous lines (Table 1), further strengthens evidence for the importance of spikelet size in determination of grain weight.

### OsINV3 Regulates Assimilate Partitioning Into the Sinks by Modulating the Hexose-to-Sucrose Ratio

The lower grain yield for the mutants, was primarily owing to the lower percentage of grain ripening and lower grain weight (Table 2). A lower grain ripening could be attributed to failure in normal development of anthers and pollen, and the process of flowering. This was consistent with the observation in *promoter::GUS* lines beginning from the early panicle development stages until after anthesis, where a strong expression of *OsINV3* in anthers (Fig. 3a-f, and Additional file 1: Figure S2D, left) and pollen (Additional file 1:Figure S1D, right), and the lodicules (Additional file 1: Figure S2C) was found. Although this prospect is co-incident with recent findings (Wang and Ruan 2016, Goetz et al. 2017), further studies are needed to delve deeper into specific role of OsINV3 in reproductive structures. While the size restriction of the grain could be stated as one of the reasons for smaller grain weight, a lower assimilate partitioning to grain was also observed (Fig. 5c), indicating an impaired transport of sucrose from source to sink tissues. In rice, sucrose transport from the source to maternal sinks along the phloem is largely believed to be symplasmic, driven by a sucrose gradient owing to the osmotic potential at the unloading site from the sieve element/companion cell complex (Aoki et al. 2012). Modifications in the sugar composition, thus the osmotic potential at the site of unloading could lead to altered capacities of sucrose import by sinks.

Hexose-to-sucrose ratio has long been considered to play a major role in modulating the osmotic gradient, thus, regulating the sink strength during the period of grain filling (Herbers and Sonnewald 1998). This ratio is found to increase during the pre-storage phase, followed by its drastic decrease, characterizing the beginning of the active storage phase of grain filling (Herbers and Sonnewald 1998). In our study, although the ratio from heading to harvest was maintained between the WT and the mutants, the ratio tended to be significantly lower in the mutants during the pre-storage phase of grain filling (Fig. 6i), with an increased sucrose concentration, which diminished the sucrose gradient along the site of unloading, facilitating a lower sucrose influx thus, a lower availability of sucrose for unloading into the filial tissues. The *promoter::GUS* studies revealed a strong *OsINV3* expression at the dorsal ovary immediately after fertilization until 6 DAF (Fig. 3g-m), serving as the critical point for directing sucrose import.

### Functional Redundancy of Vacuolar Invertase

Key biochemical functions are redundantly encoded in various isoforms to ensure normal development upon failure of one of the isoforms. Rice consists of two isoforms of vacuolar invertase, *OsINV2* and *OsINV3* (Ji et al. 2005). In the developing panicles of *OsINV3* mutants, although the development of sinks was not defective, they were reduced in size (Fig. 1f and g), owing to the lower ratios of hexose to sucrose in comparison to the WT (Fig. 6i). Lack of differences in the VIN activity between the WT and mutants (Additional file 1: Figure S5A) failed to account for the reduced hexose levels in the young panicles of the mutant (Fig. 6a). This could be attributed to the functional redundancy of VINs, where *OsINV2* was overexpressed under the absence of *OsINV3*, owing to a greater demand of hexoses in the rapidly expanding tissues; which was confirmed by an increase in mRNA transcript levels of *OsINV2* in the tissues of the *OsINV3* mutants (Additional file 1:Figure S5D). Despite this overexpression, *OsINV2* failed to substitute for the key physiological roles of *OsINV3* in maintaining the hexose-to-sucrose ratios (Fig. 6i). We suggest two possible rationale to explain this finding. First, the higher Vmax of 510 nkat mg^− 1^ for OsINV2 when compared to 20.5 nkat mg^− 1^ for OsINV3 (Ji et al. 2007), outlines a stronger possibility of the former being regulated post-translationally by inhibitors, as reported in CWINs (French et al. 2014 (in rice); Jin et al. 2009 (in tomato)). However, lack of studies on mode of regulation by VIN inhibitors renders it difficult to replicate this in in vitro assays as observed in our study (Additional file 1: Figure S5A). Second, differences in cellular or sub-cellular localization of the two isoforms could explain the inadequate substitution by OsINV2 for physiological roles of OsINV3. However, further studies need to be carried out to establish this proposition.

## Conclusion

We demonstrate a key role for a vacuolar invertase isoform, *OsINV3* in sink strength determination during the reproductive phase, mainly by regulation of grain size and assimilate partitioning to grain. *OsINV3* exercises a role in spikelet size determination mainly by regulating cell expansion, and in assimilate partitioning to grain by modulation of hexose-to-sucrose ratios during the initial phase of grain filling. In terms of assimilate partitioning and grain weight determination in cereals, by far, CWINs have been found to be key contributors. Our study attributes a similar role to VIN, mainly enabled by its regulation of spikelet size and hexose-to-sucrose ratios, and a direct influence of spikelet size on grain weights. In terms of achieving global food sustainability, attaining higher yield has been of utmost priority and our findings demonstrate a key candidate for generation of high yielding cultivars through molecular breeding studies.

## Methods

### Plant Material and Genotyping

To elucidate the physiological function of a vacuolar invertase isoform, *OsINV3*, the gene disruption lines were screened from the population of mutants carrying insertions of the *Tos17* retro-transposon, which were obtained from the National Institute of Agrobiological Sciences, Ibaraki, Japan. Detailed procedure for generation of the insertion mutants are as previously described (Miyao et al. 2003). PCR-screening was conducted using DNA pools prepared with three-dimensional sampling method from approximately 40,000 plants bearing *Tos17* insertion (Agrawal et al. 2005). Nested PCR was carried out with PrimeStar GXL DNA polymerase (Takara Bio Inc., Shiga, Japan) according to manufacturer’s instructions using combinations of primers specific to *Tos17* and *OsINV3* sequences as listed in Additional file 1:Table S1. Positive products were sequenced after gel-purification to identify the location of insertions in the rice genome. An insertion line NG6411 was isolated with a *Tos17* insertion in the second exon of *OsINV3* (Os02g0106100). The WT and mutant were segregated from the same *Tos17* insertion line NG6441 following genotyping using the primers F1 and R1 for WT allele, and F1 and T5 for the mutant allele (Additional file 1:Table S1). Genomic DNA isolation from leaves was performed using methods similar to those previously described (Okamura et al. 2013). One μl of the genomic DNA template was used for PCR with PrimeStar GXL DNA polymerase (Takara Bio Inc.) according to manufacturer’s instructions.

### Growth Conditions and Grain Size Analyses for Controlled Experiments

Seeds were sown in nursery soil in a plastic tray following chemical sterilization with 2.5% sodium hypochlorite for 30 min, an elaborate wash and imbibition with water at 30 °C for 3 days. They were placed in a growth chamber at 27 °C/22 °C, 14 h-light/10 h-dark cycle, 65% relative humidity and a light intensity of 900 μmol.m^− 2^.s^− 1^. At around 20 days after sowing, seedlings were transplanted to bigger plastic pots and grown under the same conditions.

Grain length, width and area for ten respective grains from each of the three replicate plants from WT, mutant and the T1 complement (C4) lines were determined using a digital microscope (VHX-6000, Keyence, Osaka, Japan) in concert with ImageJ 1.46r (Schneider et al. 2012).

### Generation of OsINV3 Complement Lines

The full-length ORF of *OsINV3* along with 2212 bp upstream of the putative translation start site was isolated by PCR using a high-fidelity DNA polymerase (PrimeStar GXL, Takara Bio Inc.) and primers PL2 and R7T (Additional file 1: Table S1) with genomic DNA from a rice cultivar, Nipponbare, as the template (Fig. 1b). The resultant DNA fragment was cloned into a binary vector, pZH2B (Kuroda et al. 2010) using In-Fusion HD cloning kit (Takara Bio Inc.) according to manufacturer’s instructions. The complement lines for *OsINV3* were generated by incorporation of this vector into the homozygous *Tos17* mutant using Agrobacterium (EHA105) mediated transformation (Toki 1997). Successful complementation was assessed by genotyping using F1, R1 and T5 primers (Additional file 1: Table S1). Out of the 16 complementation lines isolated, C3 and C4 were selected and propagated to T1 generation, solely based on availability of grain. Complementation tests were carried out with T2 grain from C3 and C4 showing recovery in grain size and weight characteristics, however, results are demonstrated for only the C4 line in the current study.

### SEM Analysis

Spikelet hulls for WT, mutant and C4, 2 DBH were sampled and processed as previously described (Li et al. 2011) followed by dehydration in isoamyl alcohol. The samples were dried with a critical-point drier (JEOL JCPD-5, Tokyo, Japan) and coated with Pt/Pd using a sputter coater (Hitachi E-1030, Tokyo, Japan). The SEM (Hitachi S-4800, Tokyo, Japan) was operated at 2 kV, with an aperture of 15 mm, and magnification of 120X and 200X for lemma and palea respectively. Cell density was determined as number of cells/unit area for the outer surface, and the cell area, width and height were determined for the cells on the inner surface of palea and lemma using ImageJ. Mean and SE were determined for 9 spikelet hulls for each line, with cell size analyses for 10 characteristic cells from palea and lemma of each of the 9 spikelets.

### Promoter: GUS Assay

A genomic DNA fragment that covers from − 1930 to + 30 nucleotides of the translation start site of *OsINV3* was amplified by PCR with primers PINV3-L2H and -R2X, having restriction sites HindIII and XbaI, respectively, at the 5’end of each primer (Additional file 1: Table S1). Following the double restriction digestion, the genomic DNA fragment was cloned into a binary vector, pZH2B-GUS (Kuroda et al. 2010), with a pre-existing insertion of the beta-glucoronidase (GUS) gene. Eventually, a plasmid construct, with the GUS gene flanked by 30 nucleotides downstream of the translation start site of *OsINV3* and an interconnecting XbaI (six nucleotides) site, expected to be driven by the *OsINV3* promoter was obtained (Additional file 1: Figure S1E). The GUS lines of *OsINV3* were generated by incorporation of this vector into the WT lines segregated from NG6441 using transformation techniques mentioned above. Following genotyping using PINV3-L2H and PINV3-R2X (Additional file 1: Table S1), 3 lines were isolated and propagated to T1 generation. The T1 plants that tested positive for staining were isolated and their grain used for further analyses.

Panicles at different progressive stages of panicle initiation, and through stages from heading to harvest were sampled in ice cold 90% acetone (*v*/*v*), on ice. Samples were incubated at room temperature for 20 min, followed by wash with 50 mM phosphate buffer (pH 7.0) which was repeated thrice to eliminate the acetone completely. Following this, the GUS staining was performed similar to the methods previously described (Hirose et al. 2014). Observation was carried out using a stereo microscope (SMZ745T, Nikon, Tokyo, Japan).

### Field Trials and Determination of Yield and its Components

Plants were grown in an experimental paddy field at the Institute for Sustainable Agro-ecosystem Services, The University of Tokyo (35.73°N, 139.53°E, 58 m above sea level), in Tokyo, Japan. Cultivation was carried out between the months of May and October 2015 and 2016, where one-month old seedlings were transplanted to the field on May 28, 2015 and May 25, 2016. Hills were spaced at 0.3 m × 0.15 m with one seedling per hill, leading to a planting density of 22.2 hills m^− 2^. Basal dressing of fertilizer was applied at 50 g.m^− 2^ with a N:P_2_O_5_:K_2_O of 12:16:18. Three sub-plots were maintained for each of the lines, their positions alternating with one another. Five biological replicates from each of the three sub-plots for both WT and mutant were used for assessment of yield and its components. The yield component data were obtained using methods previously described (Hirose et al. 2013).

### Dry Weight and Photosynthesis Rate Measurements

Leaves, stem, panicles and dead plant tissue from each plant were sampled separately and dried at 80 °C for at least 1 week before weighing them. Five biological replicates from each of the three sub-plots for each line were used for dry weight estimation at panicle initiation (28 DBH), heading, 10 DAH, 20 DAH and harvest (40 DAH). Total dry weight was measured on a whole plant basis by determining the sum total weights of each plant part. Dry matter partitioning to panicles or vegetative tissues was estimated on per panicle basis, as the ratio of total dry weight of panicles or vegetative tissues to the total dry weight of the plant.

Photosynthesis rates for the flag leaves of the main stem of *OsINV3* WT and the mutant (KO) were measured at panicle initiation, heading and late ripening stages using CIRAS-3 Photosynthesis Systems (PP systems, Amesbury, MA, USA) between 1100 and 1200 h. The leaf chamber was set to 30 °C, photosynthetic photon flux density of 1500 μmol.m^− 2^.s^− 1^ and CO_2_ concentration of 390 ml.L^− 1^.

### Panicle and Grain Size Analyses for Field Grown Panicles

The panicles from WT and mutant lines were harvested post-maturity (40 DAH) and air-dried for 2 weeks before analyses. The length of the panicles was measured from the tip to the base for all panicles in three replicate plants for both the lines, and the number of primary and secondary rachis branches were determined for the same sample set.

Grain length, width and area were determined for ten corresponding grains from each of the three replicate plants for both the WT and the mutant using ImageJ. For the grain thickness distribution curve, the whole grain set for three replicate plants were analyzed. The grains were made to pass through a series of sieves sized from 1.8 mm to 2.4 mm, and the grains trapped in each of these sieves were counted and weighed. The average weight per grain was obtained by the ratio of the total weight to total number of the grains corresponding the particular grain thickness.

### Quantitative Real-Time PCR

Young panicles (~ 3–4 cm in length) from WT, mutant and C4 were sampled in liquid nitrogen and pulverized cryogenically using a Multi-beads shocker (Yasui Kikai, Osaka, Japan) at 2000 rpm for 15 s. RNA isolation, cDNA synthesis and real-time PCR were performed similar to the methods previously described (Hashida et al. 2016). Primers specific to *OsINV2*, *OsINV3* and polyubiquitin gene *RUBIQ1* (Wang et al. 2000) are listed in Additional file 1:Table S1.

### Plant Sampling and Pulverization

Whole panicles from the field grown plants with two biological replicates from each of the three sub-plots for both WT and mutant, were sampled on dry ice at panicle initiation (28 DBH), heading, 5 DAH, 10 DAH, 20 DAH, 30 DAH and harvest (40 DAH). Panicle initiation was identified by the appearance of first internode at 28 DBH, where young panicles (~ 3–4 cm in length) at the base of the stem were isolated and sampled. Samples were then pulverized cryogenically as mentioned above, and weighed separately for NSC determination and the enzyme assay.

### Determination of Starch and Soluble Sugar Contents, and Invertase Enzyme Assay

NSC (Starch, sucrose, glucose and fructose) contents were determined similarly to the methods previously described (Okamura et al. 2013). Preparation of crude extract, and the soluble and insoluble enzyme assays were performed similarly to the methods previously described (Ishimaru et al. 2005), with the following modifications. The reaction buffer for acid invertase contained 100 mM of sodium acetate (pH 4.5) and 20 mM sucrose, to which 50 μl aliquot of the crude extract was added to initiate the reaction. The reaction volume was maintained at 200 μl. The reaction progressed at 37 °C for 30 min and was stopped by the addition of 20 μl of 1 M HEPES-NaOH (pH 7.5) and boiling at 98 °C for 2 min. Neutral invertase assay was performed same as above, except that the reaction buffer used contained 100 mM HEPES-NaOH (pH 7.5) instead of the acid buffer. The enzyme activity was determined as Vmax in terms of glucose generated by the reaction and was measured as described in the determination of sugar content.

### Statistical Analyses

Unpaired student’s t-test statistic (Microsoft excel 2016) was used to determine significance in differences between the WT and the mutants from minimum 3 independent biological replicates. For analyses with the complementation line, one-way ANOVA with a post-hoc analysis (Tukey’s test) was used (SPSS 13.0 for Windows). *P*-values ≤0.05, ≤ 0.01 and ≤0.001 were indicated by *, ** and *** respectively, and considered significant.

The GenBank database accession numbers for *OsINV2* and *OsINV3* used in this article are AF276703.1 and AF276704.1 respectively.

## Additional files

Additional file 1: Table S1. Details of primers used in this study. **Figure S1.** Confirmation of the presence of aberrant transcripts in the mutants (KO) using semi-quantitative PCR. **Figure S2.** Expression in the spikelet using *promoter: GUS* lines. **Figure S3.** Number of rachis branches and grain size differences between the field-grown WT and the mutants (KO). **Figure S4.** Photosynthesis rates for the WT and mutants (KO) at various growth stages from panicle initiation to late ripening. **Figure S5.** VIN, NIN and CWIN activity, and mRNA abundance of *OsINV2* in young panicles (~ 4–5 cm in length) at panicle initiation for WT and the mutants (KO). (PPTX 59980 kb)

## Abbreviations

C: Complementation
CWIN: Cell wall invertase
DAF: Days after flowering
DAH: Days after heading
DBH: Days before heading
NIN: Neutral invertase
NSC: Non-structural carbohydrates
SEM: Scanning electron microscopy
VIN: Vacuolar invertase
WT: Wild type
