# Supplementary material for: Genetic Evidence for the Role of a Rice Vacuolar Invertase as a Molecular Sink Strength Determinant
Source: Rice (N Y). 2018 Jan 17;11:6. doi: 10.1186/s12284-018-0201-x (PMC5772344; doi:10.1186/s12284-018-0201-x)
Supplement: Supplementary file 1 — Details of primers used in this study. Figure S1. Confirmation of the presence of aberrant transcripts in the mutants (KO) using semi-quantitative PCR. Figure S2. Expression in the spikelet using promoter: GUS lines. Figure S3. Number of rachis branches and grain size differences between the field-grown WT and the mutants (KO). Figure S4. Photosynthesis rates for the WT and mutants (KO) at various growth stages from panicle initiation to late ripening. Figure S5. VIN, NIN and CWIN activity, and mRNA abundance of OsINV2 in young panicles (~ 4–5 cm in length) at panicle initiation for WT and the mutants (KO). (PPTX 59980 kb) [file 12284_2018_201_MOESM1_ESM.pptx]

## Slide 1
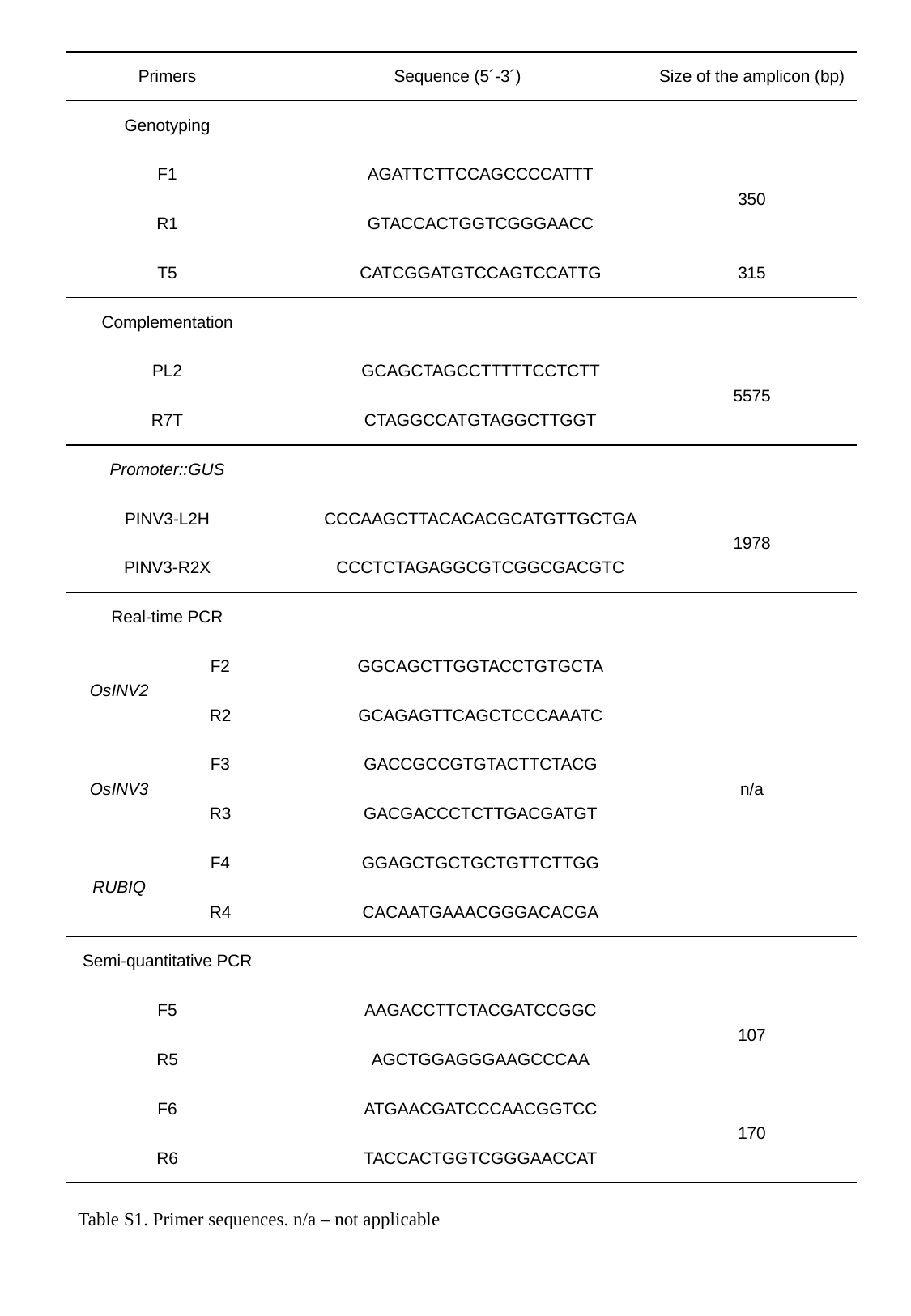

| Primers | | Sequence (5´-3´) | Size of the amplicon (bp) |
| --- | --- | --- | --- |
| Genotyping | | | |
| F1 | | AGATTCTTCCAGCCCCATTT | 350 |
| R1 | | GTACCACTGGTCGGGAACC | |
| T5 | | CATCGGATGTCCAGTCCATTG | 315 |
| Complementation | | | |
| PL2 | | GCAGCTAGCCTTTTTCCTCTT | 5575 |
| R7T | | CTAGGCCATGTAGGCTTGGT | |
| Promoter::GUS | | | |
| PINV3-L2H | | CCCAAGCTTACACACGCATGTTGCTGA | 1978 |
| PINV3-R2X | | CCCTCTAGAGGCGTCGGCGACGTC | |
| Real-time PCR | | | |
| OsINV2 | F2 | GGCAGCTTGGTACCTGTGCTA | n/a |
| | R2 | GCAGAGTTCAGCTCCCAAATC | |
| OsINV3 | F3 | GACCGCCGTGTACTTCTACG | |
| | R3 | GACGACCCTCTTGACGATGT | |
| RUBIQ | F4 | GGAGCTGCTGCTGTTCTTGG | |
| | R4 | CACAATGAAACGGGACACGA | |
| Semi-quantitative PCR | | | |
| F5 | | AAGACCTTCTACGATCCGGC | 107 |
| R5 | | AGCTGGAGGGAAGCCCAA | |
| F6 | | ATGAACGATCCCAACGGTCC | 170 |
| R6 | | TACCACTGGTCGGGAACCAT | |
Table S1. Primer sequences. n/a – not applicable

## Slide 2
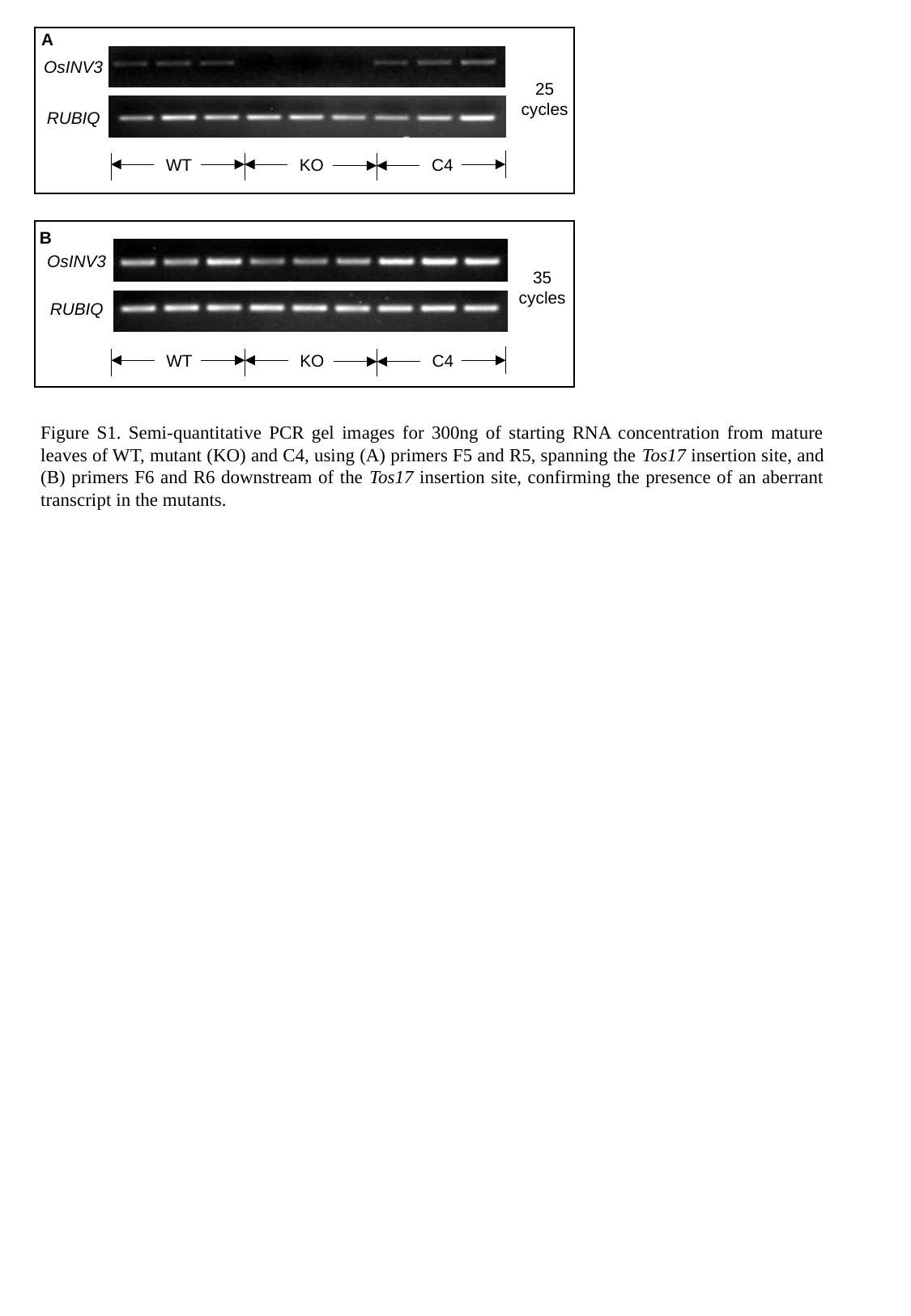

A
OsINV3
25 cycles
RUBIQ
WT
C4
KO
B
OsINV3
35 cycles
RUBIQ
WT
C4
KO
Figure S1. Semi-quantitative PCR gel images for 300ng of starting RNA concentration from mature leaves of WT, mutant (KO) and C4, using (A) primers F5 and R5, spanning the Tos17 insertion site, and (B) primers F6 and R6 downstream of the Tos17 insertion site, confirming the presence of an aberrant transcript in the mutants.

## Slide 3
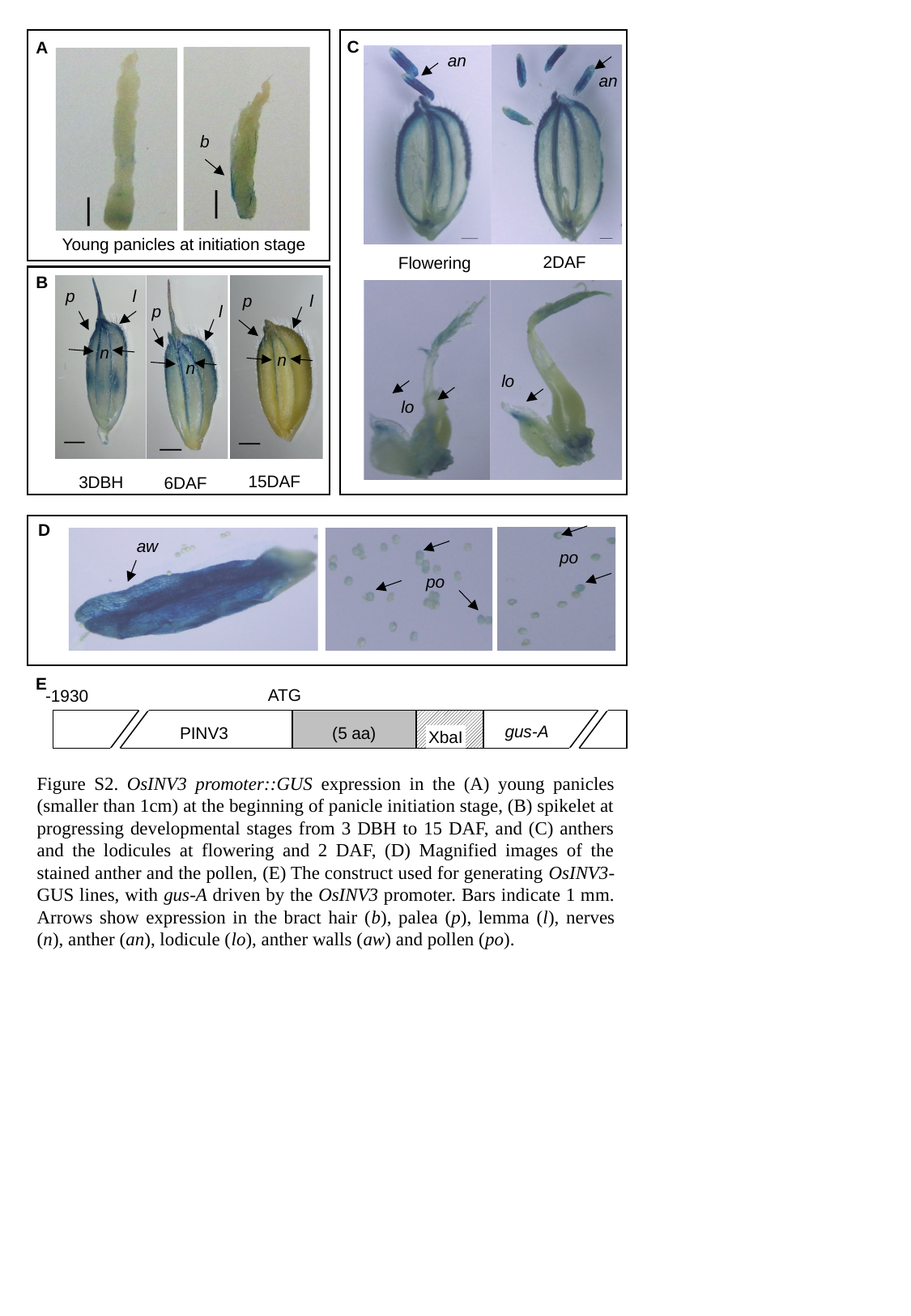

C
A
an
an
b
Young panicles at initiation stage
2DAF
Flowering
B
l
p
l
p
l
p
n
n
n
lo
lo
15DAF
3DBH
6DAF
D
aw
po
po
E
ATG
-1930
gus-A
PINV3
(5 aa)
XbaI
Figure S2. OsINV3 promoter::GUS expression in the (A) young panicles (smaller than 1cm) at the beginning of panicle initiation stage, (B) spikelet at progressing developmental stages from 3 DBH to 15 DAF, and (C) anthers and the lodicules at flowering and 2 DAF, (D) Magnified images of the stained anther and the pollen, (E) The construct used for generating OsINV3-GUS lines, with gus-A driven by the OsINV3 promoter. Bars indicate 1 mm. Arrows show expression in the bract hair (b), palea (p), lemma (l), nerves (n), anther (an), lodicule (lo), anther walls (aw) and pollen (po).

## Slide 4
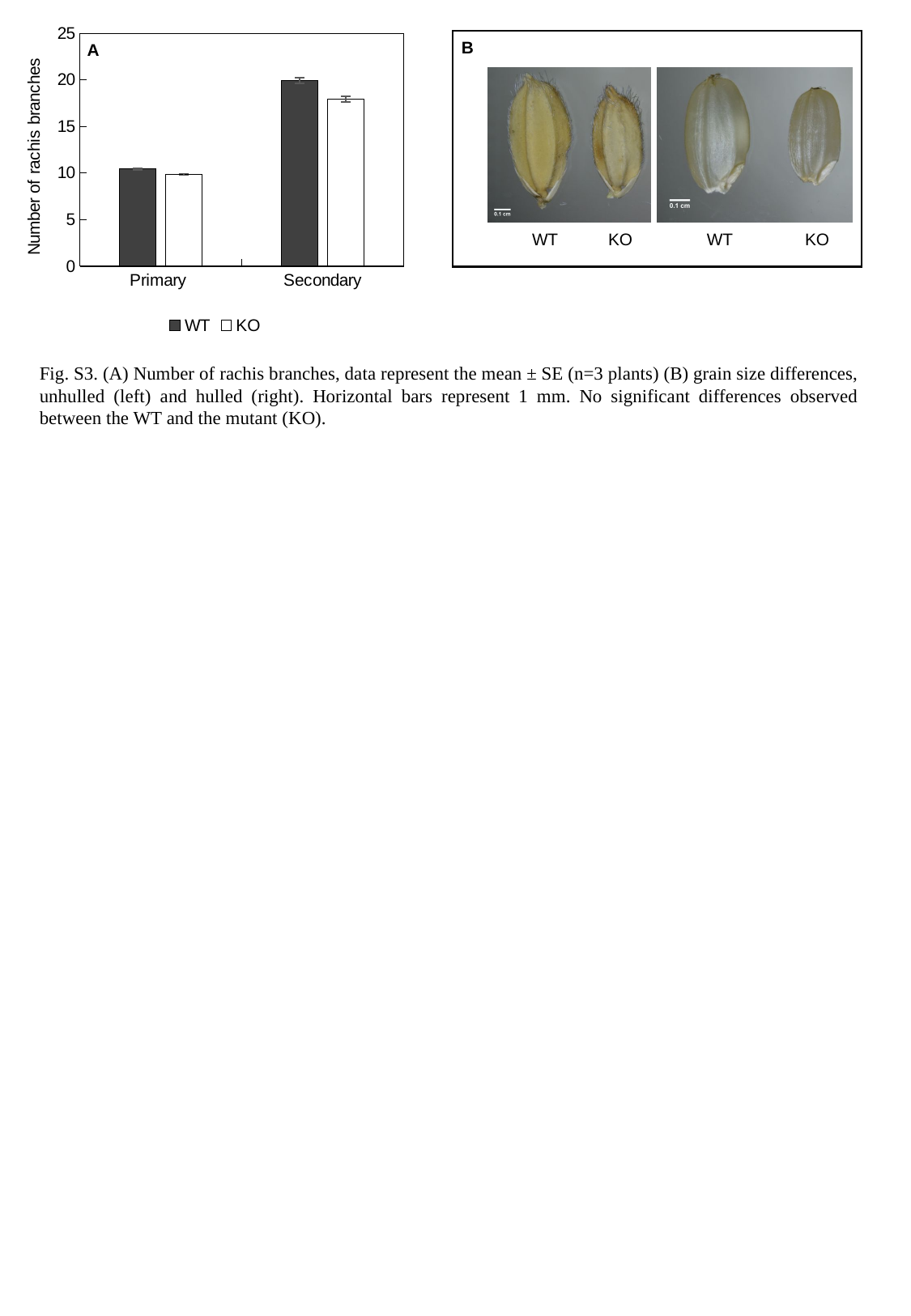

### Chart
| Category | WT | KO |
|---|---|---|
| Primary | 10.425925925925926 | 9.851851851851853 |
| Secondary | 19.925925925925927 | 17.922222222222224 |B
WT
KO
WT
KO
Fig. S3. (A) Number of rachis branches, data represent the mean ± SE (n=3 plants) (B) grain size differences, unhulled (left) and hulled (right). Horizontal bars represent 1 mm. No significant differences observed between the WT and the mutant (KO).

## Slide 5
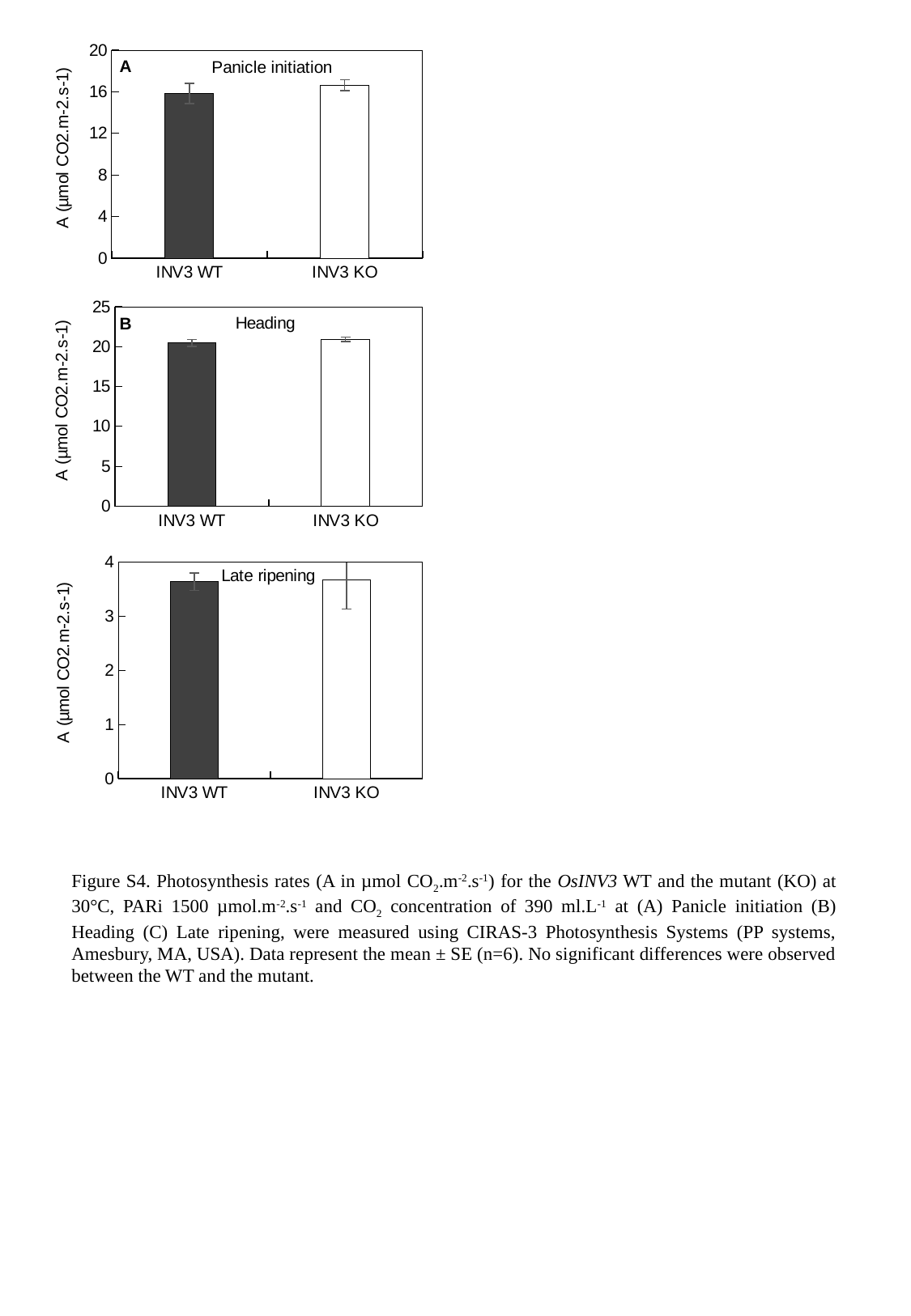

### Chart: Panicle initiation
| Category | A (µmol CO2.m-2.s-1) |
|---|---|
| INV3 WT | 15.84 |
| INV3 KO | 16.63 |A
### Chart:
| Category | Heading |
|---|---|
| INV3 WT | 20.44 |
| INV3 KO | 20.91 |
### Chart: Late ripening
| Category | 28DAH |
|---|---|
| INV3 WT | 3.64 |
| INV3 KO | 3.68 |Figure S4. Photosynthesis rates (A in µmol CO2.m-2.s-1) for the OsINV3 WT and the mutant (KO) at 30°C, PARi 1500 µmol.m-2.s-1 and CO2 concentration of 390 ml.L-1 at (A) Panicle initiation (B) Heading (C) Late ripening, were measured using CIRAS-3 Photosynthesis Systems (PP systems, Amesbury, MA, USA). Data represent the mean ± SE (n=6). No significant differences were observed between the WT and the mutant.

## Slide 6
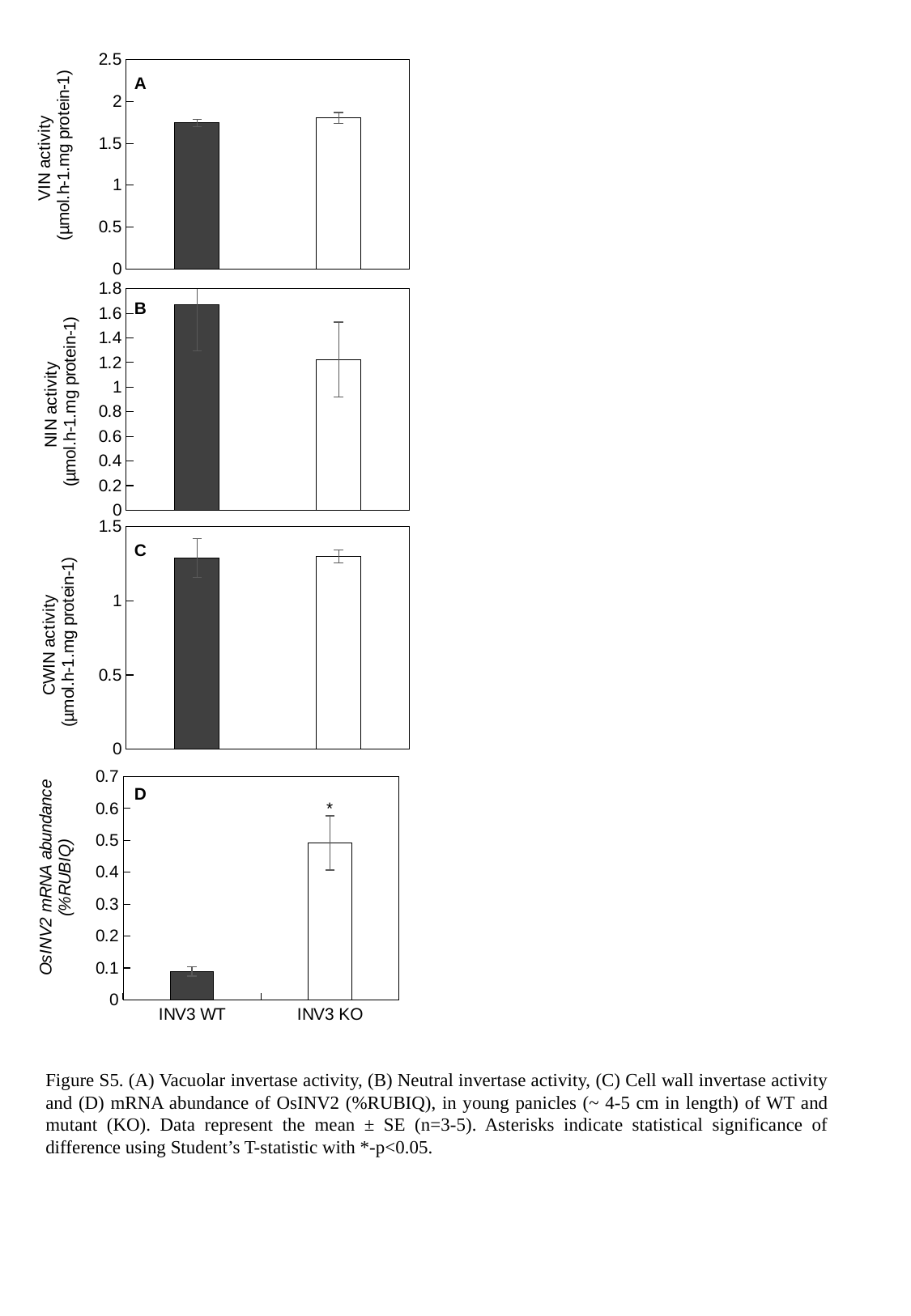

### Chart
| Category | |
|---|---|
| Inv3 WT | 1.7428745518915572 |
| Inv3 KO | 1.8043288480537316 |A
### Chart
| Category | |
|---|---|
| Inv3 WT | 1.6674966516706669 |
| Inv3 KO | 1.2247479438791051 |B
### Chart
| Category | |
|---|---|
| Inv3 WT | 1.2884230325588255 |
| Inv3 KO | 1.2992062835890579 |C
### Chart
| Category | INV2 abundance |
|---|---|
| INV3 WT | 0.08918605559133723 |
| INV3 KO | 0.49212215355267297 |D
Figure S5. (A) Vacuolar invertase activity, (B) Neutral invertase activity, (C) Cell wall invertase activity and (D) mRNA abundance of OsINV2 (%RUBIQ), in young panicles (~ 4-5 cm in length) of WT and mutant (KO). Data represent the mean ± SE (n=3-5). Asterisks indicate statistical significance of difference using Student’s T-statistic with *-p<0.05.
